# Prediction model of middle school student performance based on MBSO and MDBO-BP-Adaboost method

**DOI:** 10.3389/fdata.2024.1518939

**Published:** 2025-01-14

**Authors:** Rencheng Fang, Tao Zhou, Baohua Yu, Zhigang Li, Long Ma, Tao Luo, Yongcai Zhang, Xinqi Liu

**Affiliations:** School of Information Science and Technology, Shihezi University, Xinjiang, China

**Keywords:** feature selection, MBSO, MDBO, Adaboost, student performance prediction

## Abstract

Predictions of student performance are important to the education system as a whole, helping students to know how their learning is changing and adjusting teachers' and school policymakers' plans for their future growth. However, selecting meaningful features from the huge amount of educational data is challenging, so the dimensionality of student achievement features needs to be reduced. Based on this motivation, this paper proposes an improved Binary Snake Optimizer (MBSO) as a wrapped feature selection model, taking the Mat and Por student achievement data in the UCI database as an example, and comparing the MBSO feature selection model with other feature methods, the MBSO is able to select features with strong correlation to the students and the average number of student features selected reaches a minimum of 7.90 and 7.10, which greatly reduces the complexity of student achievement prediction. In addition, we propose the MDBO-BP-Adaboost model to predict students' performance. Firstly, the model incorporates the good point set initialization, triangle wandering strategy and adaptive t-distribution strategy to obtain the Modified Dung Beetle Optimization Algorithm (MDBO), secondly, it uses MDBO to optimize the weights and thresholds of the BP neural network, and lastly, the optimized BP neural network is used as a weak learner for Adaboost. MDBO-BP-Adaboost After comparing with XGBoost, BP, BP-Adaboost, and DBO-BP-Adaboost models, the experimental results show that the R^2^ on the student achievement dataset is 0.930 and 0.903, respectively, which proves that the proposed MDBO-BP-Adaboost model has a better effect than the other models in the prediction of students' achievement with better results than other models.

## 1 Introduction

With the introduction of big data-related research and applications in various industries, the big data industry has gained momentum in recent years. Data mining (DM) (Romero and Ventura, 2013) has tremendously helped develop fields such as IT, healthcare, and transport, tourism, and power and oil sectors (Cui et al., 2021). Additionally, DM techniques benefit the education sector, one of the areas with large amounts of data. For example, the extraction of implicit and useful educational data from a large amount of educational data contributes to predicting student performance, analyzing teaching deficiencies, and analyzing students' adaptive learning capabilities at the educational level. It can help students adjust their own learning statuses and study plans, help teachers adjust their lesson preparations according to their students' learning situations, and help schools and education policy makers design new teaching programmes (Asselman et al., 2023). Many studies have been carried out by a wide range of researchers, and in student performance prediction tasks, it is especially vital to extract data from the massive amount of available educational data that has a beneficial impact on student performance.

Yang and Li (2018) collected student educational data from 60 high schools and used Backpropagation (BP) neural networks as classification methods to predict student performance; the study showed that BP neural networks could correctly predict student performance. Shreem et al. (2022) proposed an Enhanced binary genetic algorithm (EBGA) as a wrapper selection algorithm and used five different classifiers to classify students' grades, and all of the classifiers yielded performance improvements between 1% and 10%. Yuan et al. (2024) proposed an integrated framework that combines learning behavior analysis and ML algorithms, which identifies different learning patterns of students by employing cluster analysis and uses ML algorithms to predict the performance in each pattern, and the results show that the integrated framework has a good predictive performance for the performance in the student's patterns. Bharara et al. (2018) used K-means clustering to extract the features that were most relevant to students and captured the hidden correlations between these features to improve the overall performance of the students. Turabieh et al. (2021) proposed an improved Harris hawk optimization (HHO) algorithm for discovering the most valuable features in the student performance prediction problem and used a combination of the improved HHO algorithm and a Layered recurrent neural network (LRNN) to attain 92% accuracy. Akour et al. (2020) used a model to predict the validity of student grades, which contribute to when a student will be able to complete a degree. Babu et al. (2023) used the monarch Butterfly optimization algorithm (BOA) to select features with high relevance, low complexity, and good student performance and then used Sailfish optimization (SFO) to optimize the coherence parameters of a Stacked sparse autoencoder (SSAE). Experimental tests demonstrated the effectiveness of the suggested classification model in terms of predicting students' performance, with an accuracy of 96.49%. Christou et al. (2023) wanted to predict the future performance and study time of the students by using the data from the past courses, thus collecting the data from the students in chemistry, mathematics, primary education history, philosophy and physics at the University, and proposing the FSC4RBF model for predicting the future performance of the students as well as the study time in the middle of each year, and all the experiments with regression and classification problems have yielded the best results. Asselman et al. (2023) proposes a Performance factor analysis (PFA) method based on XGBoost so as to improve the student performance prediction. It is evaluated on three student datasets, and the prediction performance is improved with the original (PFA). Although a wide range of researchers have made many contributions to big data education, it is still difficult to accurately select data features that have strong relevance to students, and the current methods achieve low classification and prediction accuracies. At the same time, student performance prediction can actively help students understand changes in their own learning situation and make timely adjustments throughout the entire education system and personalized learning systems; It can also help teachers understand students' learning status and improve their teaching work in a timely manner; It can also help school decision-makers plan the overall plan for students' learning. Therefore, in order to further handle the huge amount of data in the field of middle school, features with strong correlation with students are selected and redundant features are eliminated. This paper proposes a binary MBSO-based feature selection model, which selects features with strong correlation with students and inputs them into a BP network that is optimized by the MDBO model and uses it as a weak learner, which is integrated with Adaboost to reduce the error with respect to the actual values of the students' grades and to attain improved prediction accuracy.

The main contributions of this study are as follows:

(1) In this paper, we propose the MBSO feature selection model, which uses reflexive backward learning, variable spiral search, and golden sine strategy to improve SO and greatly reduce the possibility of the SO algorithm falling into a local optimum.
(2) A binary MBSO-based feature selection model is proposed to verify the superior performance of the MBSO model by comparing it with five feature selection algorithms, where the accuracy rate, the quantity of the selected features and the fitness value are employed as the evaluation metrics. And 7 features were selected from 32 student features, which were completely superior to the other five feature selection algorithms.
(3) The proposed Multistrategy fusion-based improved dung beetle optimization algorithm (MDBO) uses three strategies. First, a triangular wandering strategy is incorporated into a dung beetle population to reduce the likelihood of falling into local optima; second, adaptive t-distribution variability and greedy strategies are added late in the iterative process to enhance the ability of the model to jump out of local optima; and third, the triangular wandering strategy is added to the dung beetle breeding process to balance its local exploitation and global exploration capacities. Benchmarking functions are used to compare six optimization techniques, and the Wilcoxon rank sum test is used to confirm the performance of MDBO. Compared with existing methods, MDBO has stronger global search and local development capabilities, which can avoid falling into local optima and optimize relevant machine learning parameters.
(4) Based on the above research results, the MBSO feature selection model can select features with strong correlation with students, while the MDBO validated by the benchmark test functions and Wilcoxon rank sum test can accurately optimize the weights and thresholds of the BP network. After Adaboost integration, it can achieve the prediction of middle school students' grades.

The structure of the paper is as follows. The Adaboost and BP models utilized in this work are explained in Section 2. In Section 3, the enhancement provided by MBSO is explained in detail, and a binary version of MBSO is proposed as a feature selection method for choosing features from a Portuguese student dataset that are highly relevant to students. MDBO is proposed in Section 4, and its performance is evaluated using Wilcoxon's rank sum test and the results of nine benchmark test functions. In Section 5, the MDBO-BP-Adaboost model is used to conduct prediction on a Portuguese student performance dataset and to demonstrate its superior performance to that of other related models in terms of evaluation metrics. Section 6 provides a concluding summary of the entire paper.

## 2 Introduction to the model and its general framework

### 2.1 The maximum-minimum normalization

Data normalization is a common data preprocessing technique that maps data to a specific range by performing a mathematical transformation of the data, making the data comparable between different features, and the goal of data normalization is to eliminate quantitative differences in the data, making it easier to compare and analyze the data. The maximum-minimum normalization is a commonly used normalization method that maps the data linearly to the interval [0, 1], thus making the data easier to analyze and operate.

### 2.2 The BP model

BP is a multilayer feedforward model with input, hidden, and output layers that is typically utilized for supervised learning applications (Wang et al., 2015). Each neuron accepts the input from the previous layer and calculates a weighted sum, which is converted by the activation function before being output to the subsequent layer. Along with capabilities such as self-learning and self-adaptation, the model computes the error between the expected and actual outputs, which is then sent backwards through the network via backpropagation. The weights are also updated based on the contribution of each neuron to the error using the chain rule. Multiple iterations are used to decrease the error value and make the network output value close to the desired actual output value.

### 2.3 The XGBoost model

XGBoost is a powerful gradient boosting algorithm that is widely used in reality for classification and regression tasks. Its main idea is to improve the predictive performance of a model by combining multiple weak learners. Its core idea is to train a weak model first, and then adjust the training process of the subsequent models according to the wrong prediction of that model, so as to gradually reduce the prediction error. XGBoost has good and efficient performance and scalability, and reduces overfitting by controlling the complexity of the model through early stopping strategy and regularization. Therefore, it is chosen as the baseline model in this paper.

### 2.4 Adaboost algorithm

Adaboost is an integrated learning algorithm that improves the predictive performance of a model by minimizing policy probabilities (Zhao et al., 2023). Through iterative training, the weight of the next weak learner is computationally adjusted based on the last prediction error value, and then the weights of the given samples are dynamically adjusted according to the weight of the weak learner so that the next weak learner pays more attention to the samples with large prediction differences. Ultimately, weighting is used to merge several weak learners into a strong learner that provides robust performance, has great generalizability, and is better able to address gradient explosion and overfitting issues than other models.

### 2.5 Student performance prediction modeling frameworks

**Figure 1:**
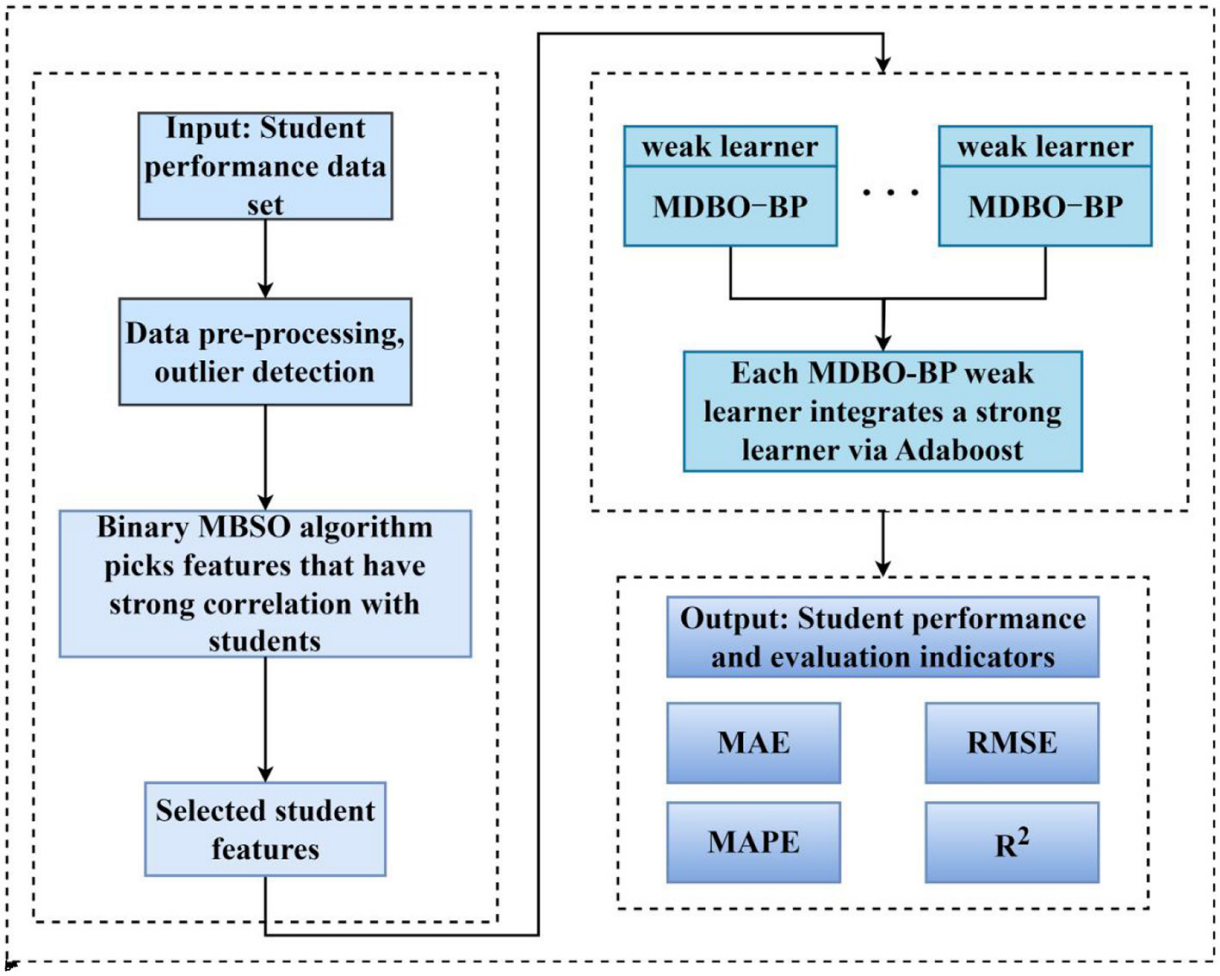
Model framework diagram.

The MDBO-BP-Adaboost model is proposed to predict student performance; this approach includes processing a student performance dataset and selecting features from the student dataset using a binary MBSO algorithm. The subset of features selected by the binary MDBO algorithm, which have high relevance, low complexity, and good student performance, are input into the BP model, which is then optimized by the MDBO algorithm to form a weak learner for integration with Adaboost. The framework diagram of the MDBO-BP-Adaboost model is shown in Figure 1.

## 3 Binary MBSO-based feature selection

### 3.1 Snake optimizer

Optimization algorithms have always been moving forward (Yang et al., 2024), and the use of a Snake optimizer (SO) (Hashim and Hussien, 2022) is mainly based on the tendency of snakes to mate under low-temperature and food-sufficient conditions, and the SO process can be divided into two phases: exploration and exploitation. All the individuals are divided into females and males, and the position update formulas of the two populations are exactly the same throughout the algorithmic process.

#### 3.1.1 Population initialization

As with all optimization algorithms, the SO requires the generation of uniformly distributed random populations, enabling the optimization process to be carried out, and the individual position initialization model is as follows:


(1) $$\begin{array}{l} X_{i}=X_{\min}+rand\times \left(X_{\max}-X_{\min}\right) \end{array}$$

*X*_max_ and *X*_min_ are the maximum and minimum values of the problem being solved, and take a random value at [0,1] is selected and assigned to *rand*.

The environmental temperature coefficients *T*_*emp*_ and food quantities *Q* associated with snake activity are displayed below:


(2) $$\begin{array}{l} \{\begin{array}{c} T_{emp}=\exp\left(-t/T\right)\\ Q=c_{1}*\exp\left[\left(t-T\right)/T\right] \end{array} \end{array}$$

The division of exploration and exploitation during SO search is controlled by food quantity *Q* and temperature *T*_*emp*_.

When *Q* < 0.25, the algorithm is in the exploratory phase, where males search for food by moving their positions, their positions are updated via the following equation:


(3) $$\begin{array}{l} X_{i,m}\left(t+1\right)=X_{rand,m}\left(t\right)\pm c_{2}\times A_{m}\times \\ \left[\left(X_{\max}-X_{\min}\right)\times rand+X_{\min}\right] \end{array}$$

A value of 0.05 is assigned to *c*_2_, and *A*_*m*_ is the food seeking capacity of a male, which it is computed by the following formula:


(4) $$\begin{array}{l} A_{m}=\exp\left(\frac{-f_{rand,m}}{f_{i,m}}\right) \end{array}$$

*f*_*rand,m*_ is the random fitness value for male individuals, and *f*_*i,m*_ is the fitness value for male search agents.

When *Q* ≥ 0.25, the algorithm enters the exploitation phase. In this phase, when *T*_*emp*_ > 0.6, the males exploit the area near the food, and males only move in the direction of the food. Their positions are updated as follows:


(5) $$\begin{array}{l} X_{i,m}\left(t+1\right)=X_{food}\left(t\right)\pm c_{3}\times T_{emp}\times \\ \left(X_{food}\left(t\right)-X_{i,m}\left(t\right)\right)\times rand \end{array}$$

*X*_*food*_(*t*) is the position of the food at iteration *t*, and a value of 2 is assigned to *c*_3_.

For *Q* ≥ 0.25 and *T*_*emp*_ ≤ 0.6, males choose either the fighting mode or the mating mode for positional updating purposes based on the randomly generated probability *p* ∈ [0, 1].

If *p* > 0.6, males and females select the fighting mode according to Equation 6, otherwise, they select the mating mode according to Equation 7, as shown below:


(6) $$\begin{array}{l} X_{i,m}\left(t+1\right)=X_{i,m}\left(t\right)\pm c_{3}\times F_{m}\times rand\\ \times \left[Q\times X_{best,f}\left(t\right)-X_{i,m}\left(t\right)\right] \end{array}$$

(7) $$\begin{array}{l} X_{i,m}\left(t+1\right)=X_{i,m}\left(t\right)\pm c_{3}\times M_{m}\times rand\\ \times \left[Q\times X_{i,f}\left(t\right)-X_{i,m}\left(t\right)\right] \end{array}$$

Where *X*_*best,f*_(*t*) is the optimal position of a female individual at the *t*_th_ iteration and *F*_*m*_ and *M*_*m*_ are the fighting and mating abilities of a male individual at position *X*_*i,m*_(*t*), respectively. The associated formulas are shown as follows:


(8) $$\begin{array}{l} \{\begin{array}{c} F_{m}=\exp\left(\frac{-f_{best,f}}{f_{i}}\right)\\ M_{m}=\exp\left(\frac{-f_{i,f}}{f_{i,m}}\right) \end{array} \end{array}$$

The random quantity *egg* ∈ {−1, 1} determines whether the mating process is successful; if *egg* = 1, mating is successful, and the male individual *X*_*worst,m*_(*t*) with the largest fitness value is updated to the following position:


(9) $$\begin{array}{l} X_{worst,m}\left(t\right)=X_{\min}+rand\times \left(X_{\max}-X_{\min}\right) \end{array}$$

### 3.2 Improved SO

According to the “there is no such thing as a free lunch” theorem (Wolpert and Macready, 1997), the SO has the drawback of eventually sliding into local optima at a later stage of the optimization process despite its great optimization accuracy and quick convergence when solving optimization problems. Therefore, the following improvement measures are suggested to increase the accuracy of the SO when solving particular problems.

#### 3.2.1 Refractive reverse learning strategy

**Figure 2:**
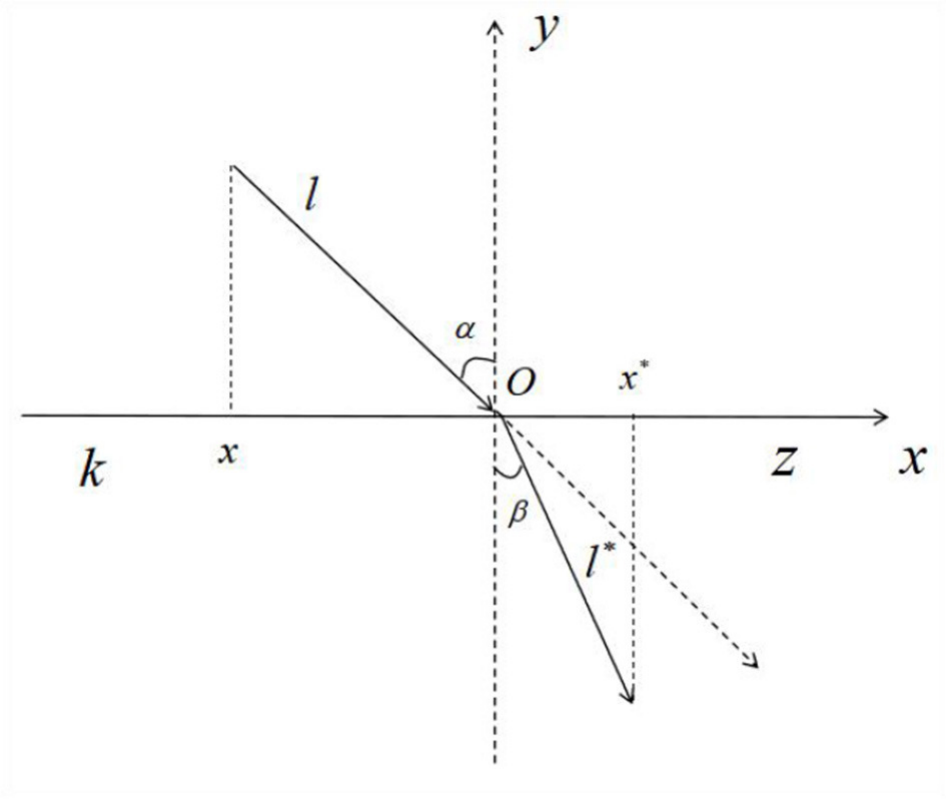
Refractive reverse learning.

The SO easily falls into local optimal solutions in the later part of the optimization search process. A refractive reverse learning mechanism is used to expand the search space for both male and female snake individuals. The search range is expanded by calculating the inverse solution of the current solution to determine a better alternative solution for the given problem. At the same time, to solve the problem of the SO easily falling into a local optimum in the late stage of reverse learning, a refraction mechanism is integrated into reverse learning. Figure 2 illustrates the primary idea of this strategy.

where the solution range on the *x* − *axis* is [*k, z*], the *y* − *axis* is the normal direction in the refractive inversion process, α and β denote the incidence and refraction angles, respectively, *l* and l^*^ denote the lengths corresponding to the incident and refracted rays, respectively, and *O* is the origin. The associated formulas are shown below:


(10) $$\begin{array}{l} \{\begin{array}{c} \text{sin }\alpha =\left(\left(k+z\right)/2-x\right)/l\\ \text{sin }\beta =\left(x^{*}-\left(k+z\right)/2\right)/l^{*} \end{array} \end{array}$$

The refractive index formula is defined as *n* = sinα/sinβ, which gives the following refractive index formula:


(11) $$\begin{array}{l} n=\frac{l^{*}\left(\left(k+z\right)/2-x\right)}{l\left(x^{*}-\left(k+z\right)/2\right)} \end{array}$$

By substituting the scaling factor *k* = *l*/l^*^, *n* = 1 into Equation 11 and generalizing it within the high-dimensional SO space, the following equation is obtained:


(12) $$\begin{array}{l} {x^{*}}_{i,j}=\frac{k_{j}+z_{j}}{2}+\frac{k_{j}+z_{j}}{2k}-\frac{x_{i,j}}{k} \end{array}$$

*x*_*i,j*_ is the position of the *i*_th_ snake individuals in the *j* dimension of the population, and ${x^{*}}_{i,j}$ is the refractive inverse position of *x*_*i,j*_.

#### 3.2.2 Variant spiral search strategy

A spiral search approach is incorporated into the position update formulation for the SO exploration phase, drawing inspiration from the whale optimization algorithm (WOA) (Mirjalili and Lewis, 2016). The spiral search formula of the WOA is a fixed helix (Chang et al., 2023). This paper proposes an improved variable spiral search strategy to adjust the shape of the helix during the search process as the iterations proceed and to enhance the exploration capabilities of individual snake males; the specific formulas for doing so are given below:


(13) $$\begin{array}{l} \beta =e^{kl}\times \cos\left(2\pi k\right) \end{array}$$

(14) $$\begin{array}{l} l=e^{2\cos\left(\frac{t}{T}\pi \right)} \end{array}$$

Where *l* progressively changes with the quantity of repetitions, a random value within [0, 1] is selected and assigned to *k*, and the cosine function controls the spiral. As the iterative process proceeds, the spirals gradually change from large to small, searching for targets with larger spiral shapes in the early stage of the algorithm, searching for as many better individuals as possible, and enhancing the global search capability of the SO. This strategy also reduces the quantity of ineffective searches in the later stage of the iterative process by searching for targets with small spiral shapes to improve the optimization search accuracy and convergence efficiency of the algorithm. The following is the new position update equation:


(15) $$\begin{array}{l} X_{i,m}(t+1)=X_{rand,m}(t)\pm \beta \times c_{2}\times A_{m}\\ \times \left[(X_{\max}-X_{\min})\times rand+X_{\min}\right] \end{array}$$

#### 3.2.3 Golden sine strategy

To force male individuals to deviate from a local optimum, a golden sine method is presented in this study. To obtain a potentially better search region, this strategy reduces the solution space via the golden section coefficient and forces the sinusoidal function to traverse all positions within the circular search range in accordance with the angular relationship between the unit circle and the sinusoidal function. Utilizing the golden sine technique, the location of the male individual from the previous iteration is updated. The formula for updating a position is displayed below:


(16) $$\begin{array}{l} X_{i,m}\left(t+1\right)=X_{i,m}\left(t\right)\times |\sin\left(r_{1}\right)|-r_{2}\times \sin|r_{1}|\\ \times |r_{3}X_{pos}-r_{4}X_{i,m}\left(t\right)| \end{array}$$

A random value within [0, 2π] is selected and assigned it to *r*_1_ and *r*_2_, and *r*_3_ and *r*_4_ are the golden section coefficients, whose expressions are displayed below:


(17) $$\begin{array}{l} \{\begin{array}{c} r_{3}=a\tau +b\left(1-\tau \right)\\ r_{4}=a\left(1-\tau \right)+b\tau \end{array} \end{array}$$

where $\tau =\left(\sqrt{5}-1\right)/2\approx 0.6183$, *a* takes the value of −π and *b* takes the value of π .

### 3.3 Binary MBSO for feature selection

#### 3.3.1 Binary MBSO

Feature selection is a method that reduces data from high to low dimensions, and the combination of the encapsulation-based feature selection method with optimization algorithms can effectively reduce the quantity of data dimensions and further improve the accuracy and efficiency of data classification results (Mostafa et al., 2023; Houssein et al., 2024). Therefore, in this paper, we convert MBSO to binary MBSO, describe the search space in a binary form and use the K-nearest neighbors (KNN) classifier to evaluate the metrics yielded by the obtained features (Arora and Anand, 2019). The binary MBSO process is described as follows.

In the initialization phase of the algorithm, a set of 0, 1 vectors are randomly generated through Equation 18. During the iteration process, the updated snake population individuals are converted into binary vectors through Equation 19.


(18) $$\begin{array}{l} X_{i,j}=\{\begin{array}{cc} 1 & X_{i,j}>0.5\\ 0 & X_{i,j}\leq 0.5 \end{array} \end{array}$$

(19) $$\begin{array}{l} X_{i,j}^{t+1}=\{\begin{array}{cc} 1 & rand<S\left(X_{i,j}^{t+1}\right)\\ 0 & others \end{array} \end{array}$$

(20) $$\begin{array}{l} S\left(X_{i,j}^{t+1}\right)=\frac{1}{1+e^{-X_{i,j}^{t+1}}} \end{array}$$

#### 3.3.2 Fitness function

Fitness functions are typically used to assess the quality of each solution during the iterative procedure of an algorithm. An outstanding classification outcome is obtained when there are few feature selection subsets, a low average fitness value, and a high classification accuracy. Therefore, the quantity of the selected feature subsets is selected based on the classification accuracy and features of the solution obtained by the KNN classifier (where K = 5). The designed fitness function is shown below:


(21) $$\begin{array}{l} Fitness=\alpha \times error+\beta \left(1-R/N\right) \end{array}$$

Where *error* is the classification error rate; *R* and *N* are the quantity of features selected by the binary MDBO feature selection process and the quantity of features that have not undergone feature selection, respectively; and 0.9 is assigned to parameter α. The parameter β = 1 − α is the importance of the selected features.

**Figure 3:**
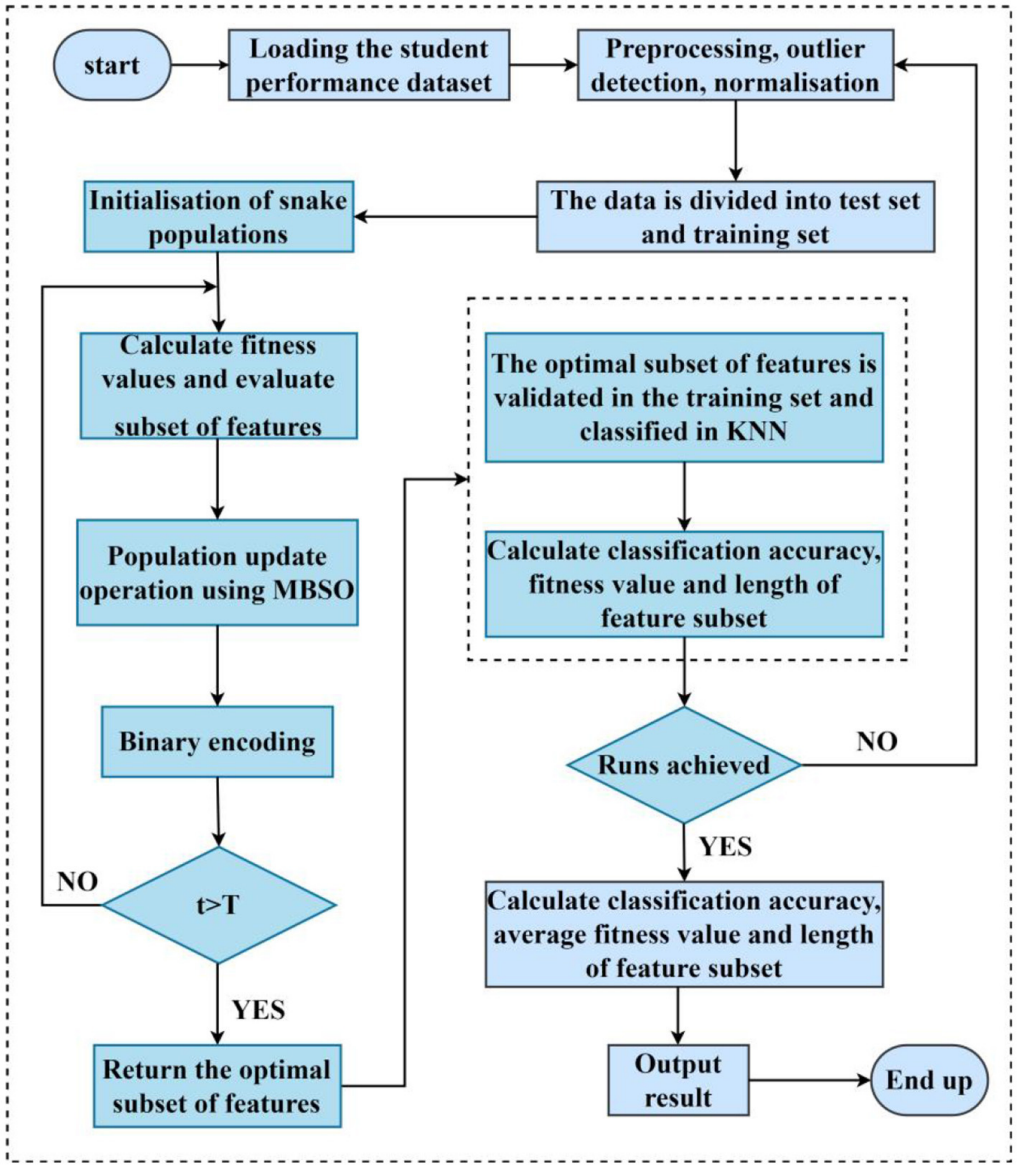
Binary MBSO for feature selection.

Figure 3 is the overall flowchart of the binary MBSO for feature selection.

## 4 Multistrategy fusion-based improved dung beetle optimizer

### 4.1 Dung beetle optimizer

The DBO classifies beetles into four subpopulations, rolling, breeding, foraging, and stealing groups, with a strong optimality-seeking ability and fast convergence (Xue and Shen, 2023). The DBO model is described below.

#### 4.1.1 Rolling dung beetles

Because light intensity affects the travel of a dung beetle and the sun is required for it to continue rolling a dung ball along a straight path in the absence of obstacles, the changes of the location of *X* is shown in the following equation:


(22) $$\begin{array}{l} X_{i}\left(t+1\right)=X_{i}\left(t\right)+a\times k\times X_{i}\left(t-1\right)+b\times \Delta X \end{array}$$

(23) $$\begin{array}{l} \Delta X=|X_{i}\left(t\right)-X^{w}| \end{array}$$

where *k* ∈ (0, 0.2] denotes the deflection coefficient, a random value within (0,1) is selected and assigned to *b*, *a* is associated with either −1 or 1, Δ*X* is the variation in the light intensity, and the global worst position is represented by *X*^*W*^.

The position update equation for an obstruction encountered by a dung beetle is displayed below:


(24) $$\begin{array}{l} X_{i}\left(t+1\right)=X_{i}\left(t\right)+\tan\left(\theta \right)|X_{i}\left(t\right)-X_{i}\left(t-1\right)| \end{array}$$

θ ∈ [0, π], but when θ equals 0, π/2, or π, the position of the dung beetle is not updated.

#### 4.1.2 Breeding dung beetle

A dung beetle will select an appropriate location to lay its eggs after rolling its dung ball back to a safe location. Therefore, a boundary selection strategy is proposed to model the region where female dung beetles deposit their eggs:


(25) $$\begin{array}{l} \begin{array}{c} Lb^{*}=\max\left(X^{*}\times \left(1-R\right),Lb\right)\\ Ub^{*}=\min\left(X^{*}\times \left(1-R\right),Ub\right) \end{array} \end{array}$$

where Lb^*^ and Ub^*^ denote the lower and upper boundaries of the spawning area, respectively, and *R* = 1 − *t*/*T*_max_.

The positions of breeding dung beetles are dynamic during the iterative process since a female will select a point in the spawning area once it is established. The changes of the location of breeding dung beetle is shown in the following equation:


(26) $$\begin{array}{l} X_{i}\left(t+1\right)=X^{*}+b_{1}\times \left(B_{i}\left(t\right)-Lb^{*}\right)+b_{2}\times \left(B_{i}\left(t\right)-Ub^{*}\right) \end{array}$$

*b*_1_ and *b*_2_ denote two independent random 1 × *D* vectors, and *D* is the dimension of the optimization problem.

#### 4.1.3 Foraging dung beetles

The optimal foraging zones must be determined when the juvenile dung beetles hatch to direct them toward food sources. The borders of these regions are displayed below:


(27) $$\begin{array}{l} \begin{array}{c} Lb^{b}=\max\;\left(X^{b}\times \left(1-R\right),Lb\right)\\ Ub^{b}=\min\;\left(X^{b}\times \left(1-R\right),Ub\right) \end{array} \end{array}$$

where the global best position is represented by *X*^*b*^. The following formula can be used to update the location of a juvenile dung beetle once its optimal feeding region has been identified:


(28) $$\begin{array}{l} x_{i}\left(t+1\right)=x_{i}\left(t\right)+C_{1}\times \left(x_{i}\left(t\right)-Lb^{b}\right)+C_{2}\times \left(x_{i}\left(t\right)-Ub^{b}\right) \end{array}$$

#### 4.1.4 Stealing dung beetles

A few dung beetles obtain their food from other dung beetles. From Equation 27, the neighborhood of *X*^*b*^ is a good representation of the finest place to compete for food. Thus, the following equation describes the process of updating the location of a stealing dung beetle:


(29) $$\begin{array}{l} x_{i}\left(t+1\right)=X^{b}+S\times g\times \left(\left|x_{i}\left(t\right)-X^{*}|+|x_{i}\left(t\right)-X^{b}\right|\right) \end{array}$$

*g* is a random vector with a size of 1 × *D* that obeys a normal distribution, and S denotes a constant.

### 4.2 The proposed MDBO algorithm

The DBO has issues with its limited global search ability and its propensity to settle for local optima. To improve the ability of the DBO to conduct local exploitation, and conduct global searches, MDBO is proposed.

#### 4.2.1 Good point set initialization strategy

The dung beetle population is initialized by the DBO in a randomly dispersed manner, which makes it difficult to obtain a uniformly distributed population. To increase the accuracy and convergence speed of the DBO, we use a good point set strategy to initialize the dung beetle population in this paper, which allows the initial dung beetle population to be spread more evenly (Hua and Wang, 2012). Additionally, the population of the good generated dung beetle point set is denoted as *P* and is described by the following equation:


(30) $$\begin{array}{l} P_{n}\left(k\right)=\left\{\left(\left\{r_{1}^{\left(n\right)}\times k\right\},\left\{r_{2}^{\left(n\right)}\times k\right\},...,\left\{r_{s}^{\left(n\right)}\times k\right\},1\leq k\leq n\right)\right\} \end{array}$$

Where *P*_*n*_(*k*) is the set of good points, *s* is the dimensionality, *r* denotes the good points, and the value of the set of good points *r* is taken as:


(31) $$\begin{array}{l} r=\left\{2\cos\left(2\pi k/p\right),1\leq k\leq s\right\} \end{array}$$

*p* is the smallest prime quantity that satisfies the condition (*p* − 3)/2 ≥ *s*. Therefore, the new initialization strategy is:


(32) $$\begin{array}{l} x_{i}\left(j\right)=\left(ub_{j}-lb_{j}\right)\times \left\{r_{j}^{i}\times k\right\}+lb_{j} \end{array}$$

**Figure 4:**
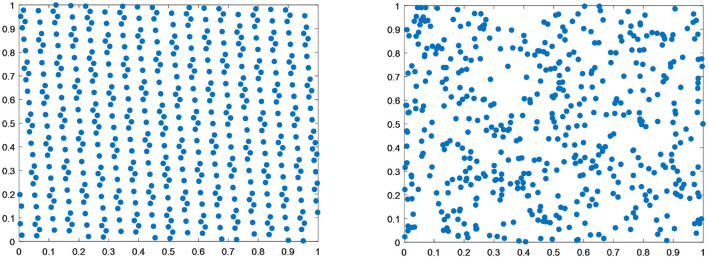
Comparison between the good point set initialization and random initialization distributions.

Figure 4 shows that the distribution produced by the good point set initialization process is more uniform than that of random initialization.

#### 4.2.2 Triangle wandering strategy

The introduction of a triangular wandering strategy for breeding dung beetles, who do not need to be directly close to the optimal spawning area but instead wander around the spawning area, allows the algorithm to have a better local search ability in later stages. First, the distance between a dung beetle and the spawning area is obtained as *L*_1_. Then, the range of the walking step length of the dung beetle is obtained as *L*_2_. Where *L*_1_ and *L*_2_ are shown in Equations 33, 34, and the walking direction β of the dung beetle is obtained according to Equation 35. Then, the distance *P* between the current location of the dun beetles and the breeding area is calculated according to Equation 36. Finally, the position of the dung beetle after implementing the triangular wandering strategy is obtained from Equation 37.


(33) $$\begin{array}{l} L_{1}=pos_{b}\left(t\right)-pos_{c}\left(t\right) \end{array}$$

(34) $$\begin{array}{l} \vec{L_{2}}=rand()\times \vec{L_{1}} \end{array}$$

(35) $$\begin{array}{l} \beta =2\times \pi \times rand() \end{array}$$

(36) $$\begin{array}{l} P=L_{1}+L_{2}-2\times L_{1}\times L_{2}\times \cos\left(\beta \right) \end{array}$$

(37) $$\begin{array}{l} P\text{o}{\text{s}}_{\text{new}}=p\text{o}{\text{s}}_{\text{b}}\left(t\right)+r\times P \end{array}$$

#### 4.2.3 Adaptive *t*-distribution variation and greedy strategies

The *t*-distribution contains parametric degrees of freedom *tn*, and tn varies adaptively with the quantity of iterations, which can balance the exploration and exploitation capabilities of the DBO (Wu et al., 2023). When 1 degree of freedom is used, the distribution is close to the Cauchy distribution, and as the degrees of freedom increase, the distribution gradually approaches the Gaussian distribution. Therefore, dynamically adjusting the degree-of-freedom parameters enables the DBO to improve its global search ability in the early stage to discover a wider solution space, while its local search ability is enhanced in the later stage to converge to a more accurate solution. Thus, the specific equations for updating the positions of dung beetles after implementing distribution variation are as follows.


(38) $$\begin{array}{l} trnd\left(tn\right)=\{\begin{array}{cc} G\text{auss}\left(0,1\right) & tn\to \infty \\ Cauchy\left(0,1\right) & tn=1 \end{array} \end{array}$$

(39) $$\begin{array}{l} tn=\exp\left(4.{\left(t/T\right)}^{2}\right) \end{array}$$

(40) $$\begin{array}{l} x_{i}\left(t\right)=x_{i}\left(t\right)+x_{i}\left(t\right)\times trnd\left(tn\right) \end{array}$$

*G*auss(0, 1) is the Gaussian distribution, *Cauchy*(0, 1) is the Cauchy variation, and *tn* exhibits a non-linear increase with the quantity of iterations *t*.

To further compare the position of a beetle after the mutation perturbation with the original position to see which has a better fitness value, a greedy strategy is used, and it is implemented as follows:


(41) $$\begin{array}{l} X^{p}=\{\begin{array}{cc} H^{b}\left(t\right) & f\left[H^{b}\left(t\right)\right]<f\left[X^{b}\left(t\right)\right]\\ X^{b}\left(t\right) & f\left[H^{b}\left(t\right)\right]\geq f\left[X^{b}\left(t\right)\right] \end{array} \end{array}$$

#### 4.2.4 MDBO algorithm implementation steps

**Algorithm 1:**
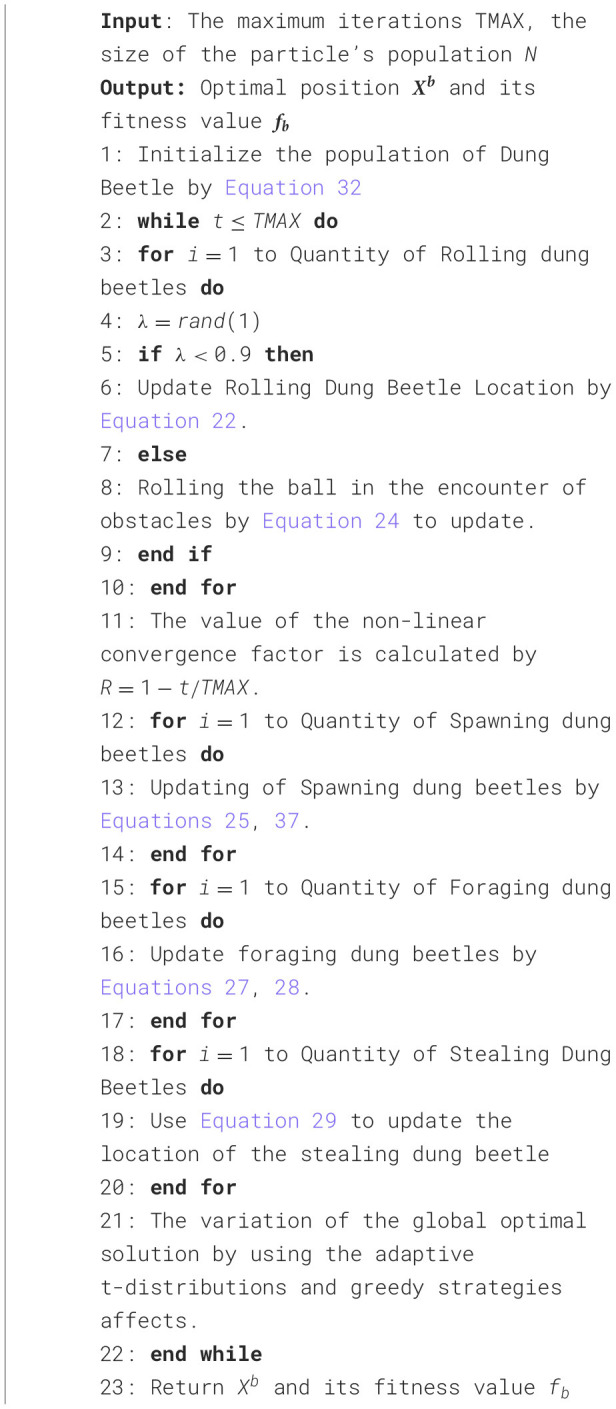
The MDBO algorithm's framework.

### 4.3 Performance of the MDBO Algorithm

#### 4.3.1 Benchmark test function experiment

To validate the performance of MDBO, the performance of the algorithm is tested and evaluated using benchmarking functions. The benchmark test functions are selected from CEC2017 (Wu et al., 2016), and these functions are shown in Table 1 (Dimensions uniformity of 30). Moreover, the parameters of the Sine cosine algorithm (SCA) (Mirjalili, 2016), WOA, HHO (Heidari et al., 2019), Golden jackal optimization (GJO) (Chopra and Ansari, 2022), SCSO, and DBO algorithm are the same as those in their original papers. For the fairness of the experiments, the initialized population sizes of all the algorithms are set to 30, and the maximum quantity of iterations is 1,000 (Jia et al., 2021). Additionally, to eliminate randomness in the experiments, 30 independent runs are made on each benchmark function, and the optimal value, mean, standard deviation and ranking of the mean are used as the evaluation metrics.

**Table 1:** Test functions.

**No**	**Types**	**Functions**	**Dim**	**Opt**
F1	Unimodal	Shifted and Rotated Bent Cigar Function	30	100
F2	Multimodal	Shifted and Rotated Zakharov Function	30	300
	F3	Shifted and Rotated Rastrigin's Function	30	500
F4	Hybrid	Hybrid function 1 (*N =* 3)	30	1,100
	F5	Hybrid function 2 (*N =* 3)	30	1,200
	F6	Hybrid function 3 (*N =* 3)	30	1,300
F7	Composition	Hybrid function 6 (*N =* 6)	30	2,000
	F8	Composition function 5 (*N =* 5)	30	2,500
	F9	Composition function 8 (*N =* 6)	30	2,800

As Table 2 illustrates, MDBO achieves excellent results on all the benchmark test functions. With respect to the optimization of the unimodal function (F1), the optimization accuracy of MDBO is closer to the theoretical optimum than that of the other six algorithms, and the proposed algorithm performs better overall. Regarding the multimodal functions (F2 and F3), the optimization accuracy achieved by MDBO for F2 is slightly lower than that of HHO, but its overall performance is better than that of the remaining five optimization algorithms, ranking second; on F3, the optimization accuracy and overall performance of MDBO are better than those of the remaining algorithms and are close to the theoretical optimal value sought for the function. For the hybrid functions (F4, F5, and F6), the optimization accuracy of MDBO is orders of magnitude greater than those of the other algorithms. On the composition function (F7), the optimization accuracy of MDBO is slightly worse than that of GJO and ranks second overall, outperforming the optimization accuracies of the remaining five optimization algorithms; its accuracy is ranked first for the F8 and F9 benchmark functions, with superior performance and accuracy to those of the remaining algorithms.

**Table 2:** Experimental results produced for the test functions.

**NO**		**SCA**	**WOA**	**HHO**	**GJO**	**SCSO**	**DBO**	**MDBO**
F1	Mean	1.86E+10	1.68E+09	2.69E+07	1.21E+10	6.05E+09	4.78E+07	**7.61E+03**
	Std	6.338E-73	7.90E+08	6.32E+06	3.38E+09	3.47E+09	7.08E+07	**7.69E+03**
	Degree	5	4	2	7	6	3	**1**
F2	Mean	6.89E+04	2.50E+05	**4.07E+04**	5.66E+04	5.00E+04	7.08E+04	4.94E+04
	Std	4.73E-49	6.60E+04	**7.00E+03**	9.63E+03	1.13E+04	1.24E+04	7.06E+03
	Degree	5	7	**1**	4	3	6	2
F3	Mean	8.19E+02	8.33E+02	7.60E+02	7.06E+02	7.55E+02	7.64E+02	**6.83E+02**
	Std	2.06E+01	5.41E+01	3.13E+01	4.28E+01	4.76E+01	4.81E+01	**5.32E+01**
	Degree	6	7	4	2	3	5	**1**
F4	Mean	3.41E+03	6.97E+03	1.30E+03	3.04E+03	2.36E+03	1.58E+03	**1.30E+03**
	Std	8.62E+02	3.26E+03	5.55E+01	1.42E+03	9.71E+02	3.68E+02	**7.74E+01**
	Degree	6	7	2	5	4	3	**1**
F5	Mean	2.06E+09	2.25E+08	2.21E+07	8.59E+08	2.78E+08	3.29E+07	**1.95E+06**
	Std	6.90E+08	1.26E+08	1.62E+07	7.56E+08	3.78E+08	4.97E+07	**3.06E+06**
	Degree	4	5	2	7	6	3	**1**
F6	Mean	8.27E+08	3.11E+06	8.45E+05	1.62E+08	4.25E+07	5.36E+06	**6.65E+04**
	Std	4.22E+08	8.12E+06	1.16E+06	2.15E+08	1.01E+08	1.37E+07	**5.81E+04**
	Degree	7	3	2	6	5	4	**1**
F7	Mean	2.85E+03	2.87E+03	2.82E+03	**2.56E+03**	2.68E+03	2.75E+03	2.65E+03
	Std	1.50E+02	1.97E+02	2.47E+02	**1.90E+02**	1.96E+02	1.63E+02	2.15E+02
	Degree	6	7	5	**1**	3	4	2
F8	Mean	3.45E+03	3.11E+03	2.94E+03	3.23E+03	3.14E+03	2.97E+03	**2.91E+03**
	Std	1.90E+02	5.54E+01	2.62E+01	1.33E+02	1.20E+02	6.27E+01	**2.56E+01**
	Degree	7	4	2	6	5	3	**1**
F9	Mean	4.29E+03	3.53E+03	3.34E+03	3.94E+03	3.60E+03	3.39E+03	**3.25E+03**
	Std	3.15E+02	1.14E+02	3.38E+01	3.78E+02	2.20E+02	1.93E+02	**4.98E+01**
	Degree	7	4	2	6	5	3	**1**

The bold value represents the minimum value of the mean.

#### 4.3.2 Wilcoxon rank sum test

Although thirty different runs are used to compare the performances of the different algorithms, additional statistical testing is still required to fully understand their capabilities. The Wilcoxon rank sum test is used to assess whether the results of each MDBO run are significantly different from those of the other algorithms at the *P* = 5\% significance level (Zhu et al., 2024). According to the null hypothesis, there should not be much difference between each pair of algorithms. *P* > 5% signifies the acceptance of the original hypothesis, implying that the two compared algorithms perform similarly; N/A indicates that the intelligent optimization algorithms perform similarly in terms of optimizing the search process and are not comparable; and *P* < 5% indicates the rejection of the original hypothesis, implying that a notable distinction is present between the two tested algorithms. The exact test results obtained for MDBO by utilizing each competing method independently are displayed in Table 3.

**Table 3:** Wilcoxon rank sum test.

	**SCA**	**WOA**	**HHO**	**GJO**	**SCSO**	**DBO**
F1	3.02E-11	3.02E-11	3.02E-11	3.02E-11	3.02E-11	1.09E-10
F2	1.55E-09	3.02E-11	2.24E-02	8.29E-06	2.51E-02	1.46E-10
F3	7.39E-11	2.15E-10	5.60E-07	5.94E-02	6.74E-06	8.84E-07
F4	3.02E-11	3.02E-11	7.06E-01	3.02E-11	1.46E-10	7.77E-09
F5	3.02E-11	3.02E-11	1.78E-10	3.02E-11	5.49E-11	2.78E-07
F6	3.02E-11	3.69E-11	3.34E-11	5.49E-11	5.19E-07	1.39E-06
F7	3.99E-04	4.46E-04	6.97E-03	9.63E-02	4.83E-01	3.78E-02
F8	3.02E-11	3.02E-11	1.53E-05	3.02E-11	3.02E-11	6.36E-05
F9	3.02E-11	6.07E-11	7.12E-09	3.34E-11	4.98E-11	4.18E-09
**+** **/-/=**	9/0/0	9/0/0	8/1/0	7/2/0	8/1/0	9/0/0

Table 3 indicates that on the F1, F2, F5, F6, F8, and F9 test functions, MDBO significantly outperforms the other six optimization algorithms; on the other hand, GJO and MDBO perform similarly well in terms of the search results obtained on F3, HHO, and MDBO perform similarly on F4, and MDBO performs similarly to GJO and SCSO on F7, with no significant differences.

By combining the findings of the Wilcoxon rank sum test with the benchmark function test conducted on CEC2017, it is found that MDBO offers notable gains in both its local and global exploration capabilities. Regarding the convergence speed, accuracy, and stability of the algorithm, MDBO performs better than the original DBO and WOA as well as other optimization algorithms. This confirms the effectiveness of the optimization technique applied in this work.

## 5 Experiment

### 5.1 Dataset

This article uses the Mat and Por student performance datasets from the UCI database as the experimental dataset. The feature attributes possessed by students in the Mat and Por datasets are the same, with a total of 33 basic student characteristics. However, there are differences in the values of specific attributes, such as exam scores, attendance rates, background information, etc. Therefore, this article introduces the main student characteristics in the Mat and por datasets, as shown in Table 4. The characteristics of the first semester grades (G1) and the second semester grades (G2) are the most important for predicting academic performance, as G1 and G2 reflect each student's previous exam scores and can be highly correlated in MBSO feature selection.

**Table 4:** Introduction to Mat and Por datasets.

**Feature name**	**Connotation**	**Range of values**
Sex	Sex of students	“F” for female or “M” for male
Age	Age of students	15 to 22
Address	Home address	“U” Urban or “R” Rural
Guardian	Student's guardian	“Mother,” “Father,” or “Other”
Schoolup	Additional education expenditure	“Yes” or “No”
Famsup	Expenditures on family education	“Yes” or “No”
Activities	Student extracurricular activities	“Yes” or “No”
Higher	Want to go to higher education	“Yes” or “No”
Famrel	Quality of family relations	From “1”-very poor to “5”-excellent
Freetime	Free time after school	From “1”-very low to “5”-very high
Absences	Number of absentees	0 to 93
G1	First semester grades	0 to 20
G2	Second semester grades	0 to 20
G3	Final score	0 to 20, Output targets

At the same time, in order to facilitate the processing of the Mat and Por datasets, the maximum-minimum normalization technique was used to make the data more standardized and easy to select features with strong correlation from student characteristics.

### 5.2 Feature selection of MBSO

**Figure 5:**
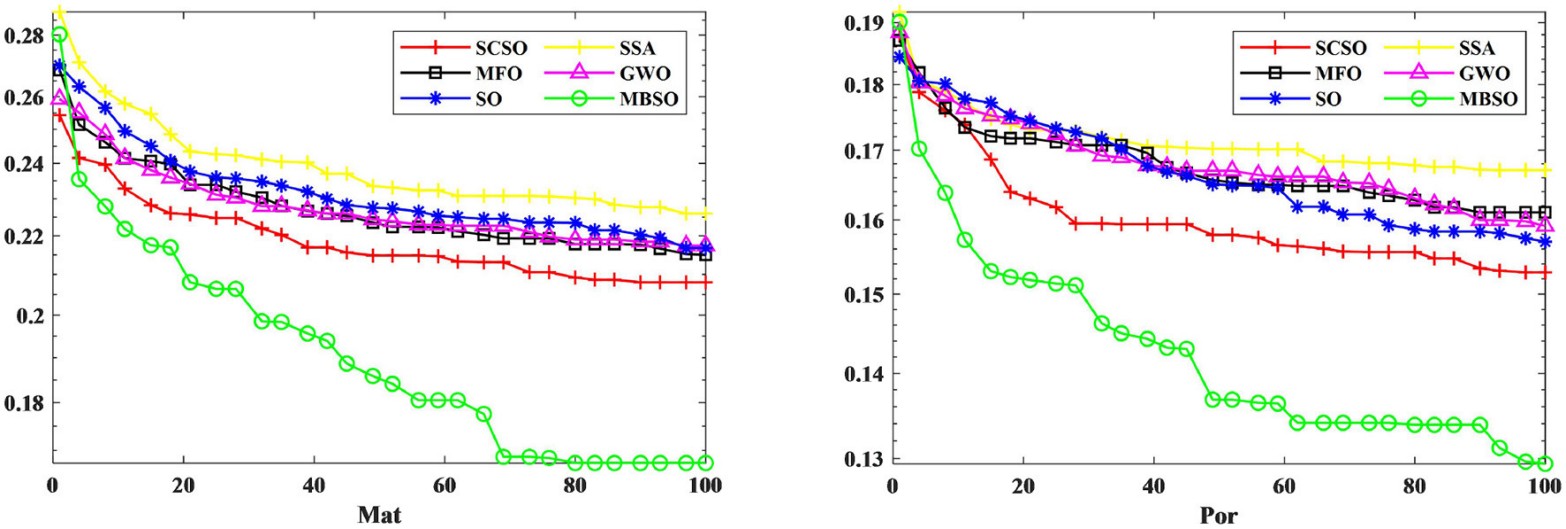
Iterative plot of adaptation for Mat and Por datasets.

In this paper, we use binary MBSO to feature select the Portuguese Mat and Por student achievement datasets and compare it with feature selection using SCSO (Seyyedabbasi and Kiani, 2023), SSA (Wang et al., 2017), MFO (Mirjalili, 2015), GWO (Mirjalili et al., 2014), SO. As can be seen from Figure 5, MBSO achieves the lowest fitness values in the dataset after 20 independent runs, while SCSO ranks second and SSA achieves the worst results. As for the number of features, it is known from Table 5 that the number of features of Mat and Por are 5.38 and 4.63, which are much lower than other feature selection methods, where in the number of Por features, MBSO is half of the number of GWO features. From Table 6, it can be concluded that MBSO feature selection is also higher than other feature selection methods in terms of accuracy, reaching 0.8414 and 0.8809, respectively.

**Table 5:** Comparison among the quantities of features chosen by MBSO and the alternative algorithms.

		**SCSO**	**SSA**	**MFO**	**GWO**	**SO**	**MBSO**
Mat	Mean	15.05	16.95	16.15	14.90	16.50	**7.90**
	Std	4.06	2.06	3.51	2.49	3.35	**5.38**
	Degree	3	6	4	2	5	**1**
Por	Mean	12.60	13.90	14.10	14.75	13.05	**7.10**
	Std	2.72	2.75	2.86	2.75	2.56	**4.63**
	Degree	2	4	5	6	3	**1**

The bold value represents the minimum value of the mean.

**Table 6:** Performance comparison of MBSO with other algorithms in terms of accuracy.

		**SCSO**	**SSA**	**MFO**	**GWO**	**SO**	**MBSO**
Mat	Mean	0.8211	0.8078	0.8172	0.8102	0.8164	**0.8414**
	Std	0.0119	0.0197	0.0125	0.0146	0.0159	**0.0356**
	Degree	2	6	3	5	4	**1**
Por	Mean	0.8739	0.8626	0.8700	0.8743	0.8709	**0.8809**
	Std	0.0140	0.0115	0.0082	0.0107	0.0107	**0.0144**
	Degree	3	6	5	2	4	**1**

The bold value represents the minimum value of the mean.

### 5.3 Score prediction of MDBO-BP- Adaboost

For the MDBO-BP-Adaboost model proposed in this paper, the optimal subset of student features obtained from the binary MBSO is firstly obtained, which is divided into the training set and test set according to 6:4. Then the MDBO algorithm is used for parameter optimization of the weights and thresholds of the BP neural network to obtain the optimal parameters, while the number of nodes in the hidden layer of the BP neural network is determined according to the empirical formulae (Wang et al., 2024), with Mat and Por being 12 and 8, respectively. and the obtained MDBO-BP is used as a weak learner (the number of weak learners is 8), which is integrated by Adaboost, and finally the proposed MDBO-BP-Adaboost student performance prediction model. The experiments were carried out on Matlab R2022a platform with lntel(R) Core(TM) i7-7500U CPU @ 2.7OGHz 2.90 GHz.

Regression evaluation measures that are frequently employed were Mean Absolute Error (MAE), Root Mean Square Error (RMSE), Mean Absolute Percentage Error (MAPE), and Coefficient of Determination (R^2^), and the importance of each evaluation indicator is the same. In this instance, the model performs better and the closer R^2^ is to 1, the better the model fits the data. The smaller the values of MAE, RMSE, and MAPE.

According to the obtained quantity of nodes input to the BP, BP-ADAboost, DBO-BP-ADAboost, MDBO-BP-ADAboost models, and combined with the XGBoost model thus resulting in the evaluation metrics for the prediction of the relevant students' performance, as shown in Tables 6, 7.

**Table 7:** Predictive effect of the model on Mat data.

**Model**	**MAE**	**RMSE**	**MAPE**	**R^2^**
XGBoost	0.944651	1.265536	0.107000	0.838
BP	0.879199	1.214926	0.083978	0.850
BP-Adaboost	0.774473	0.979547	0.075010	0.903
DBO-BP-Adaboost	0.714089	0.902263	0.070134	0.917
MDBO-BP-Adaboost	**0.654909**	**0.832686**	**0.063603**	**0.930**

The bold value represents the optimal values of MAE, RMSE, MAPE, and R2.

**Figure 6:**
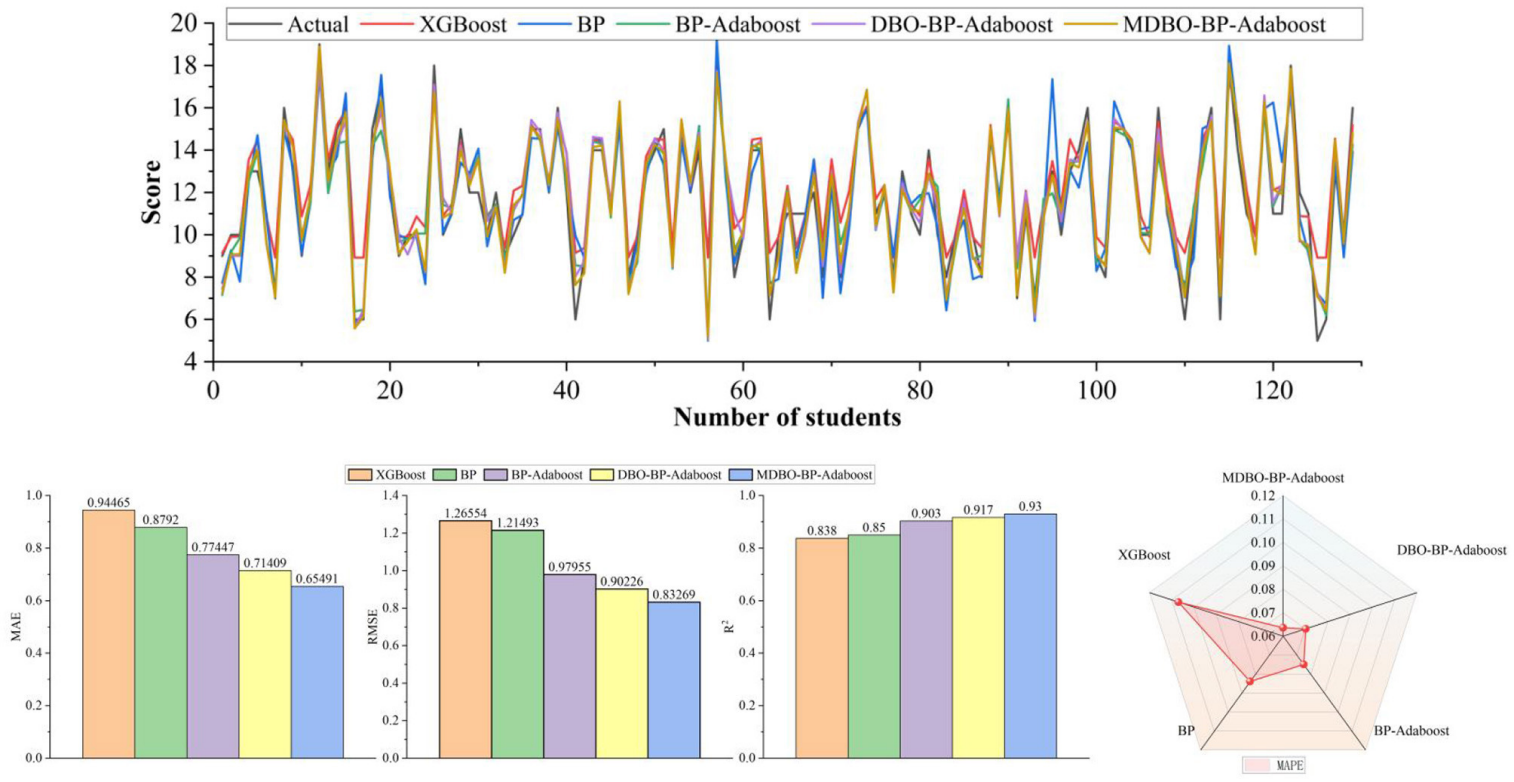
Mat dataset.

**Figure 7:**
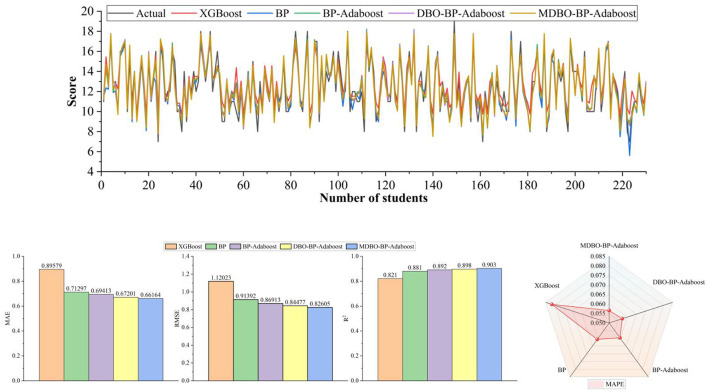
Por dataset.

As can be seen from Tables 7, 8, on the Mat student dataset, the MDBO-BP-Adaboost model proposed in this paper is compared with the XGBoost, BP, BP-Adaboost, and DBO-BP-Adaboost models, respectively, and it reduces the MAE by 30.7%, 25.5%, 15.4%, and 8.3%, RMSE by 34.2%, 31.5%, 15.0%, and 7.7%, and MAPE by 40.6%, 24.3%, 15.2%, and 9.3%, respectively, while the coefficient of determination, R^2^ was improved from 83.8% for XGBoost to 93.0% for MDBO-BP-Adaboost. Meanwhile, on the Por student dataset, the MAE decreased by 26.1%, 7.2%, 4.7%, and 1.5%, the RMSE decreased by 26.3%, 9.6%, 5.0%, and 2.2%, the MAPE decreased by 30.7%, 6.8%, 5.1%, and 1.2% while the coefficient of determination, R^2^, was improved from 0.821 for XGBoost to 0.821 for MDBO- BP-Adaboost's 0.903. It can be seen that the MDBO-BP-Adaboost grade prediction model proposed in this paper has a greater improvement in MAE, RMSE, MAPE, and R^2^ compared to other models. In terms of prediction results, the 120th student in the Mat dataset clearly shows that the predicted values of MDBO-BP-Adaboost are closer to the actual values, while the predicted values of XGBoost and BP are far from the actual values. On the Por dataset, observing students 120 to 160, it can be found that the predicted curve of MDBO-BP Adaboost is closer to the actual value, and the effect is better than that of BP Adaboost and DBO-BP Adaboost. It can be concluded that using the model in this paper for student grade prediction is more appropriate. Conversely, the curves for the Mat and Por student performance datasets in terms of projected and true values, respectively, are statistically displayed in Figures 6, 7. In the meanwhile, MDBO-BP-ADAboost performs better than the other model suggested in this research for predicting students' marks, as seen by the fact that it is closest to the true value of student grades on the predicted and true value curves.

**Table 8:** Predictive effect of the model on Por data.

**Model**	**MAE**	**RMSE**	**MAPE**	**R^2^**
XGBoost	0.895790	1.120234	0.081644	0.821
BP	0.712972	0.913917	0.060686	0.881
BP-Adaboost	0.694133	0.869131	0.059596	0.892
DBO-BP-Adaboost	0.672008	0.844774	0.057235	0.898
MDBO-BP-Adaboost	**0.661642**	**0.826050**	**0.056555**	**0.903**

The bold value represents the optimal values of MAE, RMSE, MAPE, and R2.

## 6 Conclusion

In this work, binary MBSO is presented as a feature selection method for removing data features that have minimal impacts on the predictive performance of the utilized model; lowering the likelihood of overfitting; and enhancing the generalizability, predictive accuracy, and predictive performance of the model. To assess the suitability of the proposed binary MBSO algorithm for feature selection, its performance is experimentally compared with that of five other feature selection models. The performance of the proposed binary MBSO algorithm is measured in terms of its classification accuracy, the quantity of selected feature subsets, and the fitness value produced using the KNN classifier with data acquired from two student datasets and seven UCI databases. The results demonstrate the superior performance of the proposed model by demonstrating that there is no discernible classification accuracy difference between the proposed binary MBSO approach and other algorithms, but significant improvements are observed in the average fitness value and the quantity of selected feature subsets.

The issue that the DBO tends to fall into local optima is addressed by the proposed MDBO algorithm. Moreover, the means, standard deviations, and average ranks obtained on nine fundamental test functions are compared with those of five optimization methods, and the Wilcoxon rank sum test is utilized to rank the results for assessing the effectiveness of MDBO. The aforementioned findings demonstrate the efficacy of the enhanced approach by demonstrating that MDBO outperforms the existing intelligent optimization algorithms in terms of determining the theoretically ideal values of unimodal, multimodal, hybrid, and composition functions. After passing the student dataset through the binary MBSO model feature selection, the selected subset of student features are inputted into the MDBO-BP-Adaboost model for student performance prediction and compared with other models in terms of evaluation metrics.

We validate our method on student datasets. The selected subset of student features is input into the MDBO-BP-Adaboost model to perform student performance prediction, and the results are compared with those of other models in terms of several evaluation metrics. The model reduces the MAE, RMSE, and MAPE and increases the R^2^, thereby demonstrating that the prediction results of the proposed model are more accurate than those of the competing methods. At the same time the model can be extended on other student datasets and can result in a complete student performance prediction application. Therefore, the method proposed in this paper can provide new ideas for predicting student performance, helping teachers and school policy makers analyze students' performance as well as their future learning plans.

In order to further demonstrate the practical application performance of the MDBO BP Adaboost model proposed in this paper, our next step is to obtain more student datasets from different schools, grades, and classes to verify the performance of MDBO-BP-Adaboost, in order to accurately predict students' grades and apply them to actual teaching. The model proposed in this article also has good scalability. We will preprocess the obtained student performance dataset and further obtain the basic information and past exam scores of the students to predict their grades. Furthermore, we explained the scalability of the model. At the same time, this model also has certain limitations, mainly due to the specific distribution requirements for obtaining student data, including standardization of student data and the number of student characteristics.

## Data Availability

The original contributions presented in the study are included in the article/Supplementary material, further inquiries can be directed to the corresponding author.
